# Relief and blunted defensive conditioned responses in girls with non-suicidal self-injury behaviors

**DOI:** 10.1007/s00787-026-03021-7

**Published:** 2026-04-10

**Authors:** Marta Andreatta, Nora Banaschewski, Heike Rittner, Marcel Romanos, Arne Bürger, Lorenz Deserno

**Affiliations:** 1 Department of General Psychiatry and Psychotherapy, University Hospital Tübingen, Calwerstr. 14, Tübingen, 72072 Germany; 2 German Center for Mental Health, patner site Tübingen, Tübingen, Germany; 3 Center of Mental Health, Department of Child and Adolescent Psychiatry, Psychosomatic and Psychotherapy, University Hospital Würzburg, Würzburg, Germany; 4 Centre for Interdisciplinary pain Medicine Department of Anaesthesiology, intensive Care, Emergency and Pain Medicine, University Hospital Würzburg, Würzburg, Germany

**Keywords:** Pain relief, Conditioned pain, Startle reflex, Non-suicidal self-injury, Adolescence

## Abstract

Relief is a positive emotional state that arises following the cessation of painful stimuli. Adolescents with mental health problems frequently engage in non-suicidal self-injury (NSSI). NSSI has for long been postulated as strategy for relieving intense aversive emotions. Although NSSI is hypothesized to result from a learning process, experimental evidence remains scarce. In this study, we tested whether adolescent patients suffering from NSSI have enhanced relief-learning as compared to healthy adolescents. Fifty-five girls underwent classical conditioning during which one shape (conditioned stimulus, _pain_CS) predicted a mild painful stimulation, another shape (_relief_CS) followed the pain and therefore was presented upon the moment of relief, and a third shape (CS-) was not associated with pain. Learning was quantified via physiological (startle reflex, skin conductance response) and verbal (valence, arousal and US-expectancy) responses. Adolescent patients (*N*: 28) did not differ in their relief-related responses to the healthy adolescents (*N* = 27), but they showed blunted defensive responses (lower physiological responses to _pain_CS and no discriminative verbal responses between _pain_CS and CS-). Relief-learning appears to be intact in adolescent patients with NSSI, whereas defensive responses during pain-learning were attenuated. Future studies should examine the impact of relief on instrumental learning to better elucidate the shift from healthy responses to persistent maladaptive instrumental behavior such as NSSI.

**Supplementary Information:**

The online version contains supplementary material available at https://doi.org/10.1007/s00787-026-03021-7.

## Introduction

Pain is “*an unpleasant sensory and emotional experience associated with*,* or resembling that associated with*,* actual or potential tissue damage*” [54], and has a protective function [41, 50]. Despite the aversive properties of pain, some individuals, in particular adolescents with mental problems, repeatedly elicit pain by cutting, biting, scratching or burning themselves. These behaviors are referred as non-suicidal self-injury (NSSI) that is a “*direct and deliberate destruction of body issue in absence of suicidal intent*” [51]. Typically, adolescents start this behavior at around 12 years of age [15, 49, 53] and it increases in prevalence during mid-adolescence (ages 14–16). Recent meta-analyses [15, 43] pointed out that around 10–22% of adolescents have engaged in self-injury behaviors and in females the prevalence of NSSI seem to be higher than in males, although there are regional variations [12, 49]. Notably, a history of NSSI is a robust predictor of future suicidal behaviors [12, 26, 34, 53], highlighting the clinical and social importance of better understanding its underlying mechanisms. Moreover, adults and adolescents engaging in NSSI frequently exhibit difficulties in identifying and verbalizing emotions (alexithymia), which may limit their awareness of internal states and contribute to maladaptive emotion regulation strategies [23, 52].

Currently, there are three conceptualizations on the role of pain in NSSI [57]. First, some individuals experience diminished pain perception or even pain analgesia during self-injury strategies, which may promote the repetition of NSSI behaviors [35, 51]. Over time, repeated exposure to pain may lead to habituation and individuals, consequently, engage in NSSI more frequently, because they became less sensitive to the pain [21]. Second, the experienced pain during NSSI may carry a positive value due to the regulatory effect (e.g., by distracting) over the prior emotionally negative and distressing state. In other words, being a powerful physical sensation, it diverts attention from a negative emotional state. This in turn may result in relatively more pleasant sensation, which may reinforce the self-injury behavior [32]. Third, NSSI is not linked to alterations in pain experience, but rather in the relief following a NSSI [18], a pleasant sensation experienced when pain terminates [44, 60]. The sensation of the relief is accompanied by the release of endogenous opioids [41] and by the activation of the nucleus accumbens (NAcc), a brain region involved in reward processing [7, 50]. Previous work supported this notion by showing pronounced attenuation of the startle eyeblink in adolescents engaging in NSSI 6 s after the termination of a painful electro-cutaneous stimulation [18]. This pronounced relief response, emerging with a 6-seconds delay following pain offset, aligns well with our previous findings [3], in which we examined the temporal dynamics of relief sensation and found that relief emerges with some delay following pain offset and it is experienced most strongly at 6 s after pain. The eyeblink reflex is a well-validated measure for implicit valence [9, 11, 38] and attenuation of this responses is induced by inhibitory projections from the NAcc [33, 64]. Accordingly, adolescent patients appear to respond more strongly to the termination of pain and may be more prone to repeat self-injury in order to (re-)experience the pleasant relieving feeling. Although heightened sensitivity to relief has been confirmed as a key mechanism underlying NSSI behaviors, the study by Franklin and colleagues did not rule out the involvement of additional mechanisms.

According to the opponent-process theory [60], the relieving sensation is a secondary process initiated by a painful stimulation, which reaches its peak only after the termination of this stimulation. Over repetitions, the aversiveness of the pain gradually decreases, whereas the pleasantness of the relief increases [60]. More recent theories extended this concept by suggesting that relief entails a teaching signal, which (possibly negatively) reinforces behaviors associated with pain relief [50]. Such theoretical relation between pain and its relief has been experimentally confirmed as pleasantness ratings increase and skin conductance responses (SCR) decrease following thermal pain termination [40]. Like the eyeblink startle, SCR is a broadly used index for quantifying the activation of the sympathetic nervous system [10, 38]. Interestingly, the pleasantness of the relief seems to be tightly related to the intensity of the pain meaning that the more painful the stimulation is, the stronger the relief responses become [40].

Backward conditioning is a laboratory paradigm to investigate relief-learning [14, 20, 25, 45]. During this learning protocol, an initially neutral stimulus is presented shortly after an aversive unconditioned stimulus (US, e.g. pain), specifically at the time of US termination, when a felling of relief occurs. Through backward pairing, the neutral stimulus (now labelled conditioned stimulus or CS) acquires positive properties and elicits appetitive responses [for overviews see, 19, 20]. In rodents, lesions of the NAcc [48] or depletion of the dopaminergic projections into the NAcc [46] abolished startle attenuation suggesting its crucial role in relief-learning. Human studies replicated these findings by demonstrating attenuation of the eyeblink startle [2, 4, 5], reduced SCRs [2, 5, 24] and activation of the ventral striatum, which includes the NAcc [1]. To date, these findings have been demonstrated exclusively in healthy adult populations.

Given prior evidence of enhanced relief sensitivity in adolescent patients [18] and the reinforcing effects of relief on behavior in healthy adults [19, 50], it is crucial to investigate whether adolescent patients with NSSI exhibit altered relief-related learning mechanisms considering that these patients have repeatedly experienced pain and, consequently, its relief. We hypothesized that adolescent patients with NSSI in comparisons to matched healthy adolescents would respond more pronouncedly to the stimulus associated with the relief. In other words, the relief-associated stimulus would be rated more positively and arousing by the adolescent patients compared to healthy controls, and their physiological responses (i.e., startle reflex and electro-dermal activity) would be more attenuated.

## Methods

### Participants

Seventy female volunteers participated in the study (circa half of the participants were collected before and half after the COVID19-pandemic, see supplementary material for further details). Each participant received 25€ for their participation. Exclusion criteria were being male, age below 13 years or above 18 years, insufficient knowledge of the German language, acute suicidal ideation or psychotic symptoms, and current use of psychotropic medication. Five participants were excluded due to technical problems, premature interruption of the session, or missing data in one or more conditions. Ten additional participants were defined as non-responders for the startle response (mean < 5 µV, see Data reduction), and excluded from the analyses. The final sample comprised 55 participants. Twenty-eight participants had a history of at least five times NSSI in the last year according to the DSM-5 and six of them had a comorbide borderline personality disorder (BPD)^1^. Twenty-seven participants were matched healthy controls based on age, sex and level of education (see Table 1 for descriptive statistics). The level of education was comparable for the two groups (*Χ*^2^ (7) = 12.64, *p* = 0.081; 15 participants were in high school – 10 controls and 5 patients –, 16 in secondary school – 5 controls and 11 patients –, and the remaining 9 were in different types of schools – 3 controls and 6 patients). The study was carried out in accordance with the Declaration of Helsinki (World Medical Association, 2001). The local ethic commission of the University Hospital Würzburg approved the study protocol on 05.06.2019 (36-18-me).

**Table 1:** Descriptive statistics for the two groups

	Patients	Controls	Comparisons
*N* (BPD)	28 (6)	27	
Age (*SD*)	15.86 (1.30)	15.55 (1.57)	*t*(53) = 0.80, *p* = 0.427
STAI X2 (*SD*)	61.79 (6.98)	36.52 (8.84)	*t*(53) = 11.79, *p* < 0.001
US intensity (*SD*)	2.94 (1.72) mA	2.44 (0.47) mA	*t*(53) = 1.46, *p* = 0.149
US ratings (*SD*)	6.25 (1.72)	6.65 (1.65)	*t*(53) = 0.87, *p* = 0.386

*BPD* Borderline personality disorder, *STAI X2* Trait version of the state-trait anxiety inventory, *US* Unconditioned stimulus, *SD* Standard deviation

### Stimulus material

As *unconditioned stimulus*, mildly painful electric stimuli (50 Hz, 200 ms) were administered through two electrodes to the dominant inner forearm via a constant current stimulator (Digitimer DS7A, Digitimer Ltd., Welwyn Garden City, UK). Stimulus presentation was arranged by the software Presentation (Version 1.20.0601, Neurobehavioral Systems). For individual intensity determination [for details see, 4], the initial intensity was set to 0 mA and gradually adjusted by 0.5 mA in two ascending and two descending runs. Each electrical stimulus was rated on a scale from zero (“no sensation at all”) to 10 (“very strong pain”), where 4 represents an anchor of “just noticeable pain”. Individual US intensity was aggregated as the mean of the intensities of the four runs, in which the first two intensities rated ≥ 4 for the ascending runs and the last two intensities rated ≥ 4 for the descending runs were considered. In accordance with previous study [4] and to avoid habituation, the US intensity was increased by 1 mA. The overall mean intensity was 2.69 mA (*SD*: 1.28) and was rated as painful 6.45 (*SD*: 1.69). Group comparisons can be found in Table 1.

As visual *conditioned stimuli*, we used colored geometrical shapes presented for 8 sec on a 19’’ computer screen localized circa 140 cm in front of the participants at the eye level over a black background. Shapes were a blue square, a green triangle, a yellow circle and a red hexagon with 7.8 cm width and 7.8 cm height. Shapes were counter-balanced across participants.

The *startle probe* was a white noise of 98 dB with duration of 50 ms. The acoustic stimuli were presented binaurally over headphones and occurred randomly 4–6 s after shape’s onset [11].

Participants rated the valence (“how unpleasant vs. pleasant was the stimulus?”) and the arousal (“how strong was your arousal elicited by the stimulus?”) of the visual stimuli by using two *visual analog scales* (VAS) ranging from 1 until 9. One indicates “very unpleasant” for the valence and “calm” for the arousal, while 9 indicates “very pleasant” and “exciting”, respectively. We additionally verified participants’ contingency awareness by asking them how strongly they expected the painful stimulation in association with each visual stimulus (i.e., US-expectancy ratings). The VAS ranged from 0 (never associated) until 100 (always associated).

### Questionnaires

The German version of the trait scale of the State-Trait Anxiety Inventory [STAI, 39] was collected at the beginning of the experiment. The STAI consists of 20 items, which measures the stable anxiety level of an individual (i.e., the trait). The mean score of the STAI for this sample is 49.38 (SD: 14.98) with Cronbach’s alpha 0.96 [CI95%: 0.95–0.97].

In order to control the wellbeing of the control participants, the German version of the Symptom Check List [SCL-90R, 63] was collected. This questionnaire asks about physical and mental symptoms during the last seven days on a scale ranging from 1 (not at all) to 5 (very strongly). The mean score of our sample is 0.54 (*SD*: 0.51) with Cronbach’s alpha 0.98 [CI95%: 0.96–0.99].

The short German version of the Self-Injurious Thoughts and Behavior Interview-German [SITBI-G, 16] was used in the adolescent patients. Within 72 items the SITBI-G comprises the following six constructs: suicidal thoughts, suicide plans, suicide attempts, suicidal gestures, thoughts on self-harm and actual self-harm. Each construct module begins with a screening question that clarifies whether a corresponding type has occurred in the patient. If this is the case, the section is processed with questions including age of onset, frequency (lifetime, year, month, last week), age of the first thoughts to execution, intensity at worst time point and average, chosen methods, need for medical treatment and self-reported future probabilities. Items are rated on a scale from 0 (“hardly or slightly”) to 4 (“very much or severely”).

The German version of the Positive and Negative Affect Schedule [PANAS, 37] was collected at the beginning and at the end of the experimental session to assess the actual emotional state of the participants. The PANAS consists of two scales assessing positive and negative mood by means of 10 adjectives each (all together 20 adjectives). Each adjective is rated on a 5-point Likert scale from 1 (“very slightly or not at all”) until 5 (“extremely”). The Cronbach’s alpha for the positive scale is 0.75 [CI95%: 0.57–0.86], while for the negative scale is 0.89 [CI95%: 0.81–0.94].

### Procedure

Adolescents in inpatient and outpatient wards of the Department for Child and Adolescent Psychiatry, Psychosomatics and Psychotherapy Würzburg, who fulfilled the criteria according to assessment of the ward psychologists, were approached as potential participants. The control group was derived from a participants pool of the University Hospital Würzburg as well as by distributung flyer in schools and pediatrician practices. Upon the arrival in the laboratory, participants and legal custodians read and signed the informed consent. Then, they provided sociodemographic information and filled in the questionnaires. Beforehand, participants were informed that geometrical shapes, painful electric stimulations and loud white noises will have been presented during the experiment and that they should keep the shapes in their visual focus. We did not mention the contingency between CSs and US. Afterwards, the electrodes were attached, and the pain threshold procedure was performed.

**Fig. 1:**
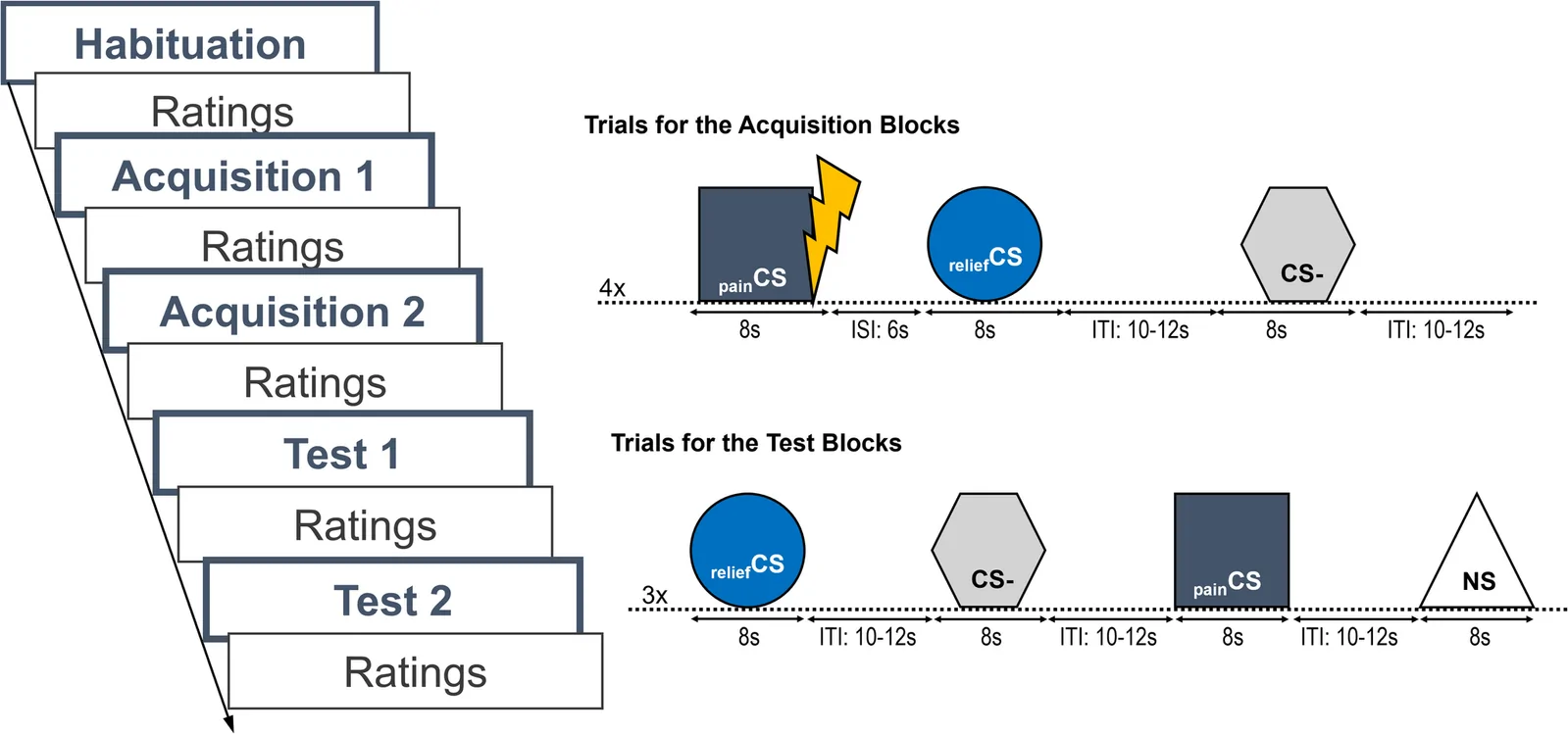
*Overview of the experimental protocol*. After a habituation block, participants underwent two acquisition blocks (Acquisition 1, Acquisition 2) followed by two blocks for test (Test 1, Test 2). Affective ratings (valence and arousal) were collected after habitation and the second acquisition block, while the US-expectancy ratings were collected after each acquisition block. All ratings were collected after each test block. During acquisition blocks, one stimulus (_pain_CS) was always followed (at the offset) by a mild painful electric stimulation (US) and six seconds later (inter-stimulus interval, ISI) a second stimulus (_relief_CS) were presented. A third stimulus (CS-) was also presented but never in association with the US. During test, the three stimuli were pseudo-randomly presented as well as a fourth additional stimulus (NS)

The experiment consisted of three phases separated by ratings (Fig. 1). For all phases, the inter-trial interval (ITI), defined as the time between stimulus offset and the subsequent stimulus onset, varied between 13 s and 18 s. For each block, a startle eliciting-probe was delivered during half of the presentations for each condition and three times during ITIs.

During the first *habituation phase*, participants passively view the four geometrical shapes on the computer screen, presented in a random order twice each. No electric stimulation or the startle probes were delivered. Shortly before the second phase, seven startle probes were randomly presented between 7 and 15 s in order to decrease the initial reactivity of the response [9]. The *acquisition phase* consisted of two identical blocks. During each block, three out of the four geometrical shapes were presented six times in a pseudo-randomized order meaning that the same condition was not presented more than twice in a row. One shape (_pain_CS) always predicted the painful electric stimulation (US), which was delivered at _pain_CS offset, while another shape (_relief_CS) was presented 6 s after the US offset [3]. The third shape (CS-) was never associated with the US. The third *test phase* also consisted of two identical blocks. The _pain_CS, the _relief_CS, and the CS- were presented five times each as well as a fourth control novel shape (NS) in a pseudo-randomized order (i.e., the same condition was not presented more than twice in a row), while the US was never delivered.

After habituation, the second acquisition block and the two test blocks, valence and arousal were collected, while US-expectancy were collected after the two acquisition and test blocks. 

### Data reduction

Physiological responses were continuously recorded with a V-Amp 16 amplifier and Vision Recorder V-Amp Edition Software (Version 1.21.0303, BrainProducts Inc., Munich, Germany). A sampling rate of 1000 Hz was applied as well as a 50 Hz notch filter. The offline analyses of these responses were conducted with Brain Vision Analyzer (Version 2.0; BrainProducts Inc., Munich, Germany).

Startle response was recorded via two 5 mm Ag/AgCl electrodes below the left eye to measure electromyographic activity of the *M. orbicularis oculi* [for guidelines see, 9]. For offline analysis, a 28 Hz low-cutoff and 400 Hz high-cutoff filter were applied. After having rectified the signal, a 50 ms moving average was used. The responses were segmented for each condition from 50 ms prior to 1 s after startle-probe onset. The 50 ms before probe onset were considered for baseline correction. Subsequently, responses were manually monitored and trials with excessive baseline shifts (≥ 5 µV, as absolute score) were excluded from analysis. The amplitude of the startle response was defined as the maximum peak between 20 and 150 ms after probe onset. If the mean startle amplitude over all trials was < 5 µV, participants were coded as non-responder and excluded from further analysis. The raw data were within-subject transformed into T-scores and averaged for each block and each stimulus (_pain_CS, _relief_CS, CS-, NS, ITI) separately. Startle responses to the visual stimuli (_pain_CS, _relief_CS, CS-, NS) were then subtracted to the ITI.

For the electrodermal activity a constant current of 0.5 V was delivered and two 8 mm Ag/AgCl electrodes were attached on the thenar and hypothenar of the non-dominant hand. We excluded for this analysis all the trials containing a startle probe, which result in six trials per condition available. The electro-dermal activity was offline filtered with 1 Hz high cutoff filter. Data were segmented 1 s before to 8 s after stimulus onset. In accordance with the guidelines [10], the SCR was defined as difference (in µS) between the response onset (0.8–4 s after shape onset) and the first peak. Responses below 0.02 µS were coded as zero and included in the analyses. After having summed 1 to the raw scores, these were then log-transformed and averaged for each condition (_pain_CS, _relief_CS, CS-, NS) and each block, separately.

### Statistical analyses

Statistical analyses were performed with the software R 3.5.1 (R Core Team, 2018). For repeated-measures analyses of variance and post-hoc *t*-test the packages afex [59] and emmeans [42] were used. The significance level was set to *p* < 0.05, partial η^2^ are reported as effect size. In case of sphericity violation, Greenhouse-Geisser correction of degrees of freedom was applied. As post-hoc tests for significant interactions, we calculated *t*-tests and the alpha level was adjusted for multiple comparisons using Bonferroni correction.

SCR, startle responses, valence, arousal, and US-expectancy ratings were separately analyzed with mixed analysis of variance (ANOVAs). For all dependent variables, ANOVAs had as between-subjects factor group (patients, controls) and as within-subject factors stimulus (ratings: _pain_CS, _relief_CS, CS-, NS; physiological responses for the acquisition: _pain_CS, _relief_CS, CS-; physiological responses for the test: _pain_CS, _relief_CS, CS-, NS) and phase. The within-subject factor phase included habituation, acquisition Block2, test Block1, and test Block2 for valence and arousal ratings, while for the US-expectancy and the physiological responses, we considered acquisition Block1, acquisition Block2, test Block1, and test Block2.

## Results

### Participant characteristics

Adolescent patients presented NSSI both thoughts and behaviors. The mean age of onset was 12.00 (*SD*: 1.51) years for the thoughts and 12.96.(*SD*: 1.53) years for the behaviors. Almost all patients (*n*: 27, 96.4%) reported suicidal thoughts, 15 patients had suicide plans (53.6%) and 12 attempted suicide (42.9%). Patients reported a high probability to repeat either the thoughts (*M*: 3.63; *SD*: 0.65), while most of them reported a high probability of possible future NSSI behaviors (*n* = 14, 50%), followed by 3 (*n* = 8, 28.6%), 2 (*n* = 3, 10.7%), 1 (*n* = 2, 7.1%) and 0 (*n* = 1, 3.6%). The mean probability to initiate a NSSI behavior was 3.14 (*SD*: 1.11). The highest intensity of the thoughts was 3.82 (*SD*: 0.39), while their intensity on average was 2.80 (*SD*: 0.70). The adolescent patients showed a great variety of NSSI frequency from 5 to 1000 times over their life span (*M*: 218.96, *SD*: 286.57) and from 5 to 200 in the last 12 months (*M*: 65.89, *SD*: 61.61). Adolescent patients self-injured themselves 0 to 40 time in a month (*M*: 6.71; *SD*: 9.77), while the week before the data collection they initiated NSSI behaviors either 0 time to 20 times (*M*: 1.50; *SD*: 3.76). The number of methods in the self-harm section ranged from 1 to 10 (*M* = 5.36 *SD* = 2.25), with the most prevalent method being cutting or carving the skin (*n* = 27, 96.4%). Further picking at a wound (*n* = 20, 71.4%), intentionally hitting oneself (*n* = 20, 71.4%), scarping the skin (*n* = 19, 67.9%), burning skin: (*n* = 14, 50%), skin chafing to the point of drawing blood (*n* = 12, 42.9%), biting oneself (*n* = 12, 42.9%), pulling out hair (*n* = 10, 37.7%), picking areas of the body to the point of drawing blood (*n* = 10, 37.7%), inserting objects under the nails or the skin (*n* = 3, 10.7%) and self-tattooing (*n* = 2, 7.1%). One part of these NSSI behaviors requested medical intervention (*n* = 11, 39.3%) as indication of the severity.

The 2 (time: beginning, end) x 2 (group: patients, controls) ANOVAs revealed significant change in mood differed between the groups (Negative Affect Scale or NAS, Time x Group: *F*(1, 53) = 12.12, *p* = 0.001, partial η^2^ = 0.186; Positive Affect Scale or PAS, Time x Group: *F*(1, 50) = 3.62, *p* = 0.063, partial η^2^ = 0.063). Adolescent patients had reduced negative mood at the end of the experiment (15.8 ± 5.64) as compared to the beginning (19.1 ± 7.08; *t*(27) = 4.39, *p* = 0.002, *d* = 0.51), while this change in mood was not evident for the healthy controls (beginning: 12.7 ± 3.34; end: 12.5 ± 4.26; *t*(26) = 0.40, *p* = 0.696, *d* = 0.05). 

### Learning effects across both groups

The learning effects as reflected in the within-subject factors stimulus and phase as well as their interaction across both groups are reported in the Supplementary Material. In summary, the learning protocol was successful across both groups as indicated by stronger defensive responses to pain-predicting cue and reward-like responses to the relief-associated cue as compared to the CS-.

### Group effects

The between-subjects factor group and its interactions with the within-subject factors stimulus and phase are reported following.

#### Ratings

The Stimulus x Phase x Group interaction was significant for the affective ratings (valence: *F*(9, 477) = 3.85, GG-ɛ = 0.592, *p* = 0.002, partial η^2^ = 0.068; arousal: *F*(9, 477) = 2.21, GG-ɛ = 0.664, *p* = 0.042, partial η^2^ = 0.040), but no significant effect involving the factor group was found for the US-expectancy (all *p* values > 0.226).

**Fig. 2:**
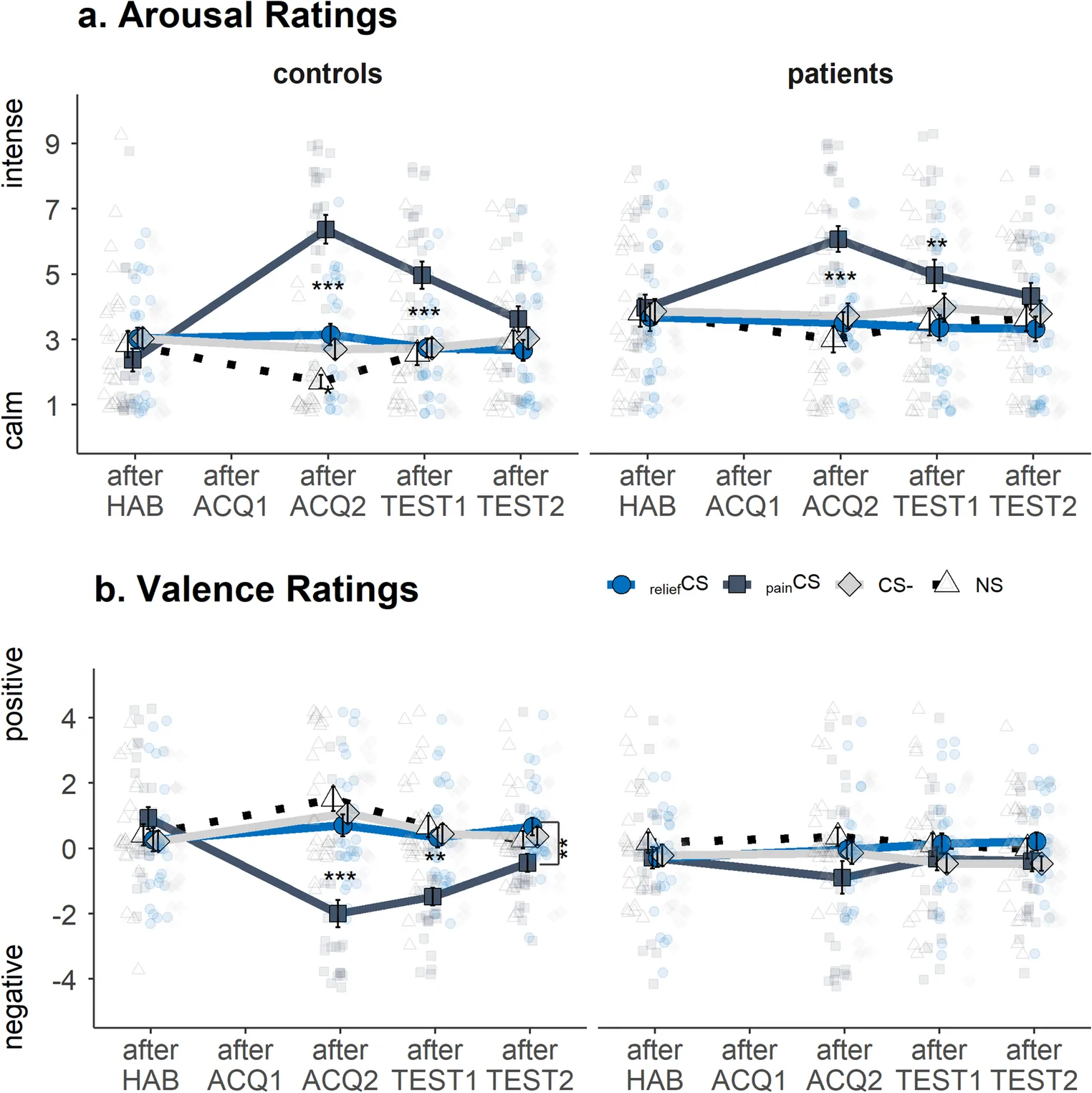
*Group differences in the verbal responses*. Lines depict means (with standard errors) for the (***a*.**) *arousal* and (***b*.**) *valence ratings* after each experimental block (habituation [HAB], first [ACQ1] and second [ACQ2] acquisition block as well as first [TEST1] and second [TEST2] test block). At baseline [HAB], no differences were found between the groups. Controls (left panels) well discriminated between _pain_CS (dark-blue lines and squares) as CS- (grey lines and diamonds), _relief_CS (light-blue lines and circles) or the control stimulus (NS, dotted lines and white triangles). In contrast, adolescent patients (right panels) showed no discriminative valence ratings and less pronounced discriminative arousal ratings *** *p* < 0.001; ** *p* < 0.01; * *p* < 0.05 (after Bonferroni correction)

Direct comparisons (Fig. 2) between the groups revealed that the CS- and the NS were more arousing (CS-: *t*(53) = 2.03, *p* = 0.048, *d* = 0.55; NS: *t*(53) = 2.82, *p* = 0.007, *d* = 0.76) and more negative (CS-: *t*(53) = 2.65, *p* = 0.011, *d* = 0.72; NS: *t*(53) = 2.50, *p* = 0.016, *d* = 0.67) for the adolescent patients than for the healthy adolescents at the end of the acquisition phase. CS- remained more arousing (first test block: *t*(53) = 2.32, *p* = 0.024, *d* = 0.63) and more negative (first test block: *t*(53) = 2.21, *p* = 0.032, *d* = 0.60; second test block: *t*(53) = 2.36, *p* = 0.022, *d* = 0.72) for the patients than for the healthy young participants throughout the test. _pain_CS was perceived less negative by the patients than by the controls at the end of the first test block (*t*(53) = 2.57, *p* = 0.013, *d* = 0.69). No other significant differences were found for the ratings (all *p* values > 0.079).

Post-hoc analyses for this significant 3-way interaction are reported in Table 2 and revealed less pronounced discriminative ratings for the adolescent patients than the healthy adolescents. Adolescent patients reported comparable valence for all stimuli after each block. Only, the _pain_CS was reported more arousing than the other cues at the end of the acquisition and remained so after the first test block compared to _relief_CS. These discriminative subjective responses quickly disappeared as no other difference was found after the second test block. Healthy adolescents rated the _pain_CS as more negative and arousing compared to the other stimuli at the end of the acquisition phase and the NS was less arousing than both the _relief_CS and the CS-. Discriminative affective ratings were maintained throughout the first test block, and extinguished at the end of the second test block.

**Table 2:** Post-hoc t-tests for the Stimulus x Phase x Group Interaction for the affective ratings

	Adolescent Patients			Healthy Adolescents		
	Acq. 2	Test 1	Test 2	Acq. 2	Test 1	Test 2
	**Valence Ratings**					
_pain_CS vs. _relief_CS	*t*(27) = 1.41, *p* > 0.999, *d* = 0.27	*t*(27) = 0.97, *p* > 0.999, *d* = 0.18	*t*(27) = 1.75, *p* = 0.546, *d* = 0.73	*** *t*(26) = 5.07, *p* < 0.001, *d* = 0.98	*** *t*(26) = 4.35, *p* = 0.001, *d* = 0.84	** *t*(26) = 3.78, *p* = 0.005, *d* = 0.73
_pain_CS vs. CS-	*t*(27) = 1.31, *p* > 0.999, *d* = 0.25	*t*(27) = 0.32, *p* > 0.999, *d* = 0.06	*t*(27) = 0.17, *p* > 0.999, *d* = 0.52	*** *t*(26) = 6.11, *p* < 0.001, *d* = 1.18	*** *t*(26) = 4.74, *p* < 0.001, *d* = 0.91	*t*(26) = 2.70, *p* = 0.073, *d* = 0.52
_pain_CS vs. NS	*t*(27) = 2.13, *p* = 0.257, *d* = 0.40	*t*(27) = 0.80, *p* > 0.999, *d* = 0.15	*t*(27) = 0.80, *p* > 0.999, *d* = 0.40	*** *t*(26) = 5.43, *p* < 0.001, *d* = 1.05	*** *t*(26) = 4.79, *p* < 0.001, *d* = 0.92	*t*(26) = 2.09, *p* = 0.280, *d* = 0.40
_relief_CS vs. CS-	*t*(27) = 0.34, *p* > 0.999, *d* = 0.06	*t*(27) = 1.73, *p* = 0.566, *d* = 0.33	*t*(27) = 1.83, *p* = 0.471, *d* = 0.27	*t*(26) = 1.33, *p* > 0.999, *d* = 0.26	*t*(26) = 0.49, *p* > 0.999, *d* = 0.09	*t*(26) = 1.40, *p* > 0.999, *d* = 0.27
_relief_CS vs. NS	*t*(27) = 0.91, *p* > 0.999, *d* = 0.17	*t*(27) = 0.07, *p* > 0.999, *d* = 0.01	*t*(27) = 0.46, *p* > 0.999, *d* = 0.22	*t*(26) = 1.77, *p* = 0.527, *d* = 0.34	*t*(26) = 1.22, *p* > 0.999, *d* = 0.24	*t*(26) = 1.16, *p* > 0.999, *d* = 0.22
CS- vs. NS	*t*(27) = 1.08, *p* > 0.999, *d* = 0.20	*t*(27) = 1.48, *p* = 0.904, *d* = 0.28	*t*(27) = 1.44, *p* = 0.974, *d* = 0.07	*t*(26) = 1.10, *p* > 0.999, *d* = 0.21	*t*(26) = 1.14, *p* > 0.999, *d* = 0.22	*t*(26) = 0.36, *p* > 0.999, *d* = 0.07
	**Arousal Rating**					
_pain_CS vs. _relief_CS	*** *t*(27) = 6.00, *p* < 0.001, *d* = 1.13	** *t*(27) = 3.65, *p* = 0.007, *d* = 0.69	*t*(26) = 2.75, *p* = 0.063, *d* = 0.52	*** *t*(26) = 6.09, *p* < 0.001, *d* = 1.17	*** *t*(26) = 5.36, *p* < 0.001, *d* = 1.03	*t*(26) = 2.80, *p* = 0.057, *d* = 0.54
_pain_CS vs. CS-	*** *t*(27) = 5.09, *p* < 0.001, *d* = 0.96	*t*(27) = 1.81, *p* = 0.486, *d* = 0.34	*t*(26) = 1.15, *p* > 0.999, *d* = 0.22	*** *t*(26) = 7.01, *p* < 0.001, *d* = 1.35	*** *t*(26) = 4.56, *p* < 0.001, *d* = 0.88	*t*(26) = 1.71, *p* = 0.597, *d* = 0.33
_pain_CS vs. NS	*** *t*(27) = 5.80, *p* < 0.001, *d* = 1.10	*t*(27) = 2.32, *p* = 0.169, *d* = 0.44	*t*(26) = 1.51, *p* = 0.851, *d* = 0.29	*** *t*(26) = 8.97, *p* < 0.001, *d* = 1.73	*** *t*(26) = 4.50, *p* = 0.001, *d* = 0.87	*t*(26) = 1.83, *p* = 0.471, *d* = 0.35
_relief_CS vs. CS-	*t*(27) = 0.53, *p* > 0.999, *d* = 0.10	*t*(27) = 1.53, *p* = 0.823, *d* = 0.29	*t*(26) = 1.02, *p* > 0.999, *d* = 0.19	*t*(26) = 1.72, *p* = 0.580, *d* = 0.33	*t*(26) = 0.00, *p* > 0.999, *d* = 0.00	*t*(26) = 2.43, *p* = 0.134, *d* = 0.47
_relief_CS vs. NS	*t*(27) = 0.99, *p* > 0.999, *d* = 0.19	*t*(27) = 0.33, *p* > 0.999, *d* = 0.06	*t*(26) = 0.64, *p* > 0.999, *d* = 0.12	** *t*(26) = 3.98, *p* = 0.003, *d* = 0.77	*t*(26) = 0.50, *p* = 0.621, *d* = 0.10	*t*(26) = 0.77, *p* > 0.999, *d* = 0.15
CS- vs. NS	*t*(27) = 1.43, *p* = 0.992, *d* = 0.27	*t*(27) = 1.01, *p* > 0.999, *d* = 0.19	*t*(26) = 0.36, *p* > 0.999, *d* = 0.07	* *t*(26) = 3.12, *p* = 0.026, *d* = 0.60	*t*(26) = 1.10, *p* = 0.284, *d* = 0.21	*t*(26) = 0.41, *p* > 0.999, *d* = 0.08

The main effect group turned out significant for arousal ratings (*F*(1, 53) = 5.02, *p* = 0.029, partial η^2^ = 0.087), while for valence ratings just failed to reach the significance level (*F*(1, 53) = 3.59, *p* = 0.064, partial η^2^ = 0.063) suggesting higher arousal (*M*: 3.91; *SD*: 2.22) and slightly lower valence (*M*: 4.84; *SD*: 1.70) ratings for the adolescent patients as compared to the healthy adolescents ([arousal] *M*: 3.16; *SD*: 2.04; [valence] *M*: 5.25; *SD*: 1.83).

In summary, adolescent patients suffering from NSSI behaviors rated the pain-associated stimulus with lower arousal and valence resulting in reduced pain-related defensive learning.

#### Physiological responses

**Fig. 3:**
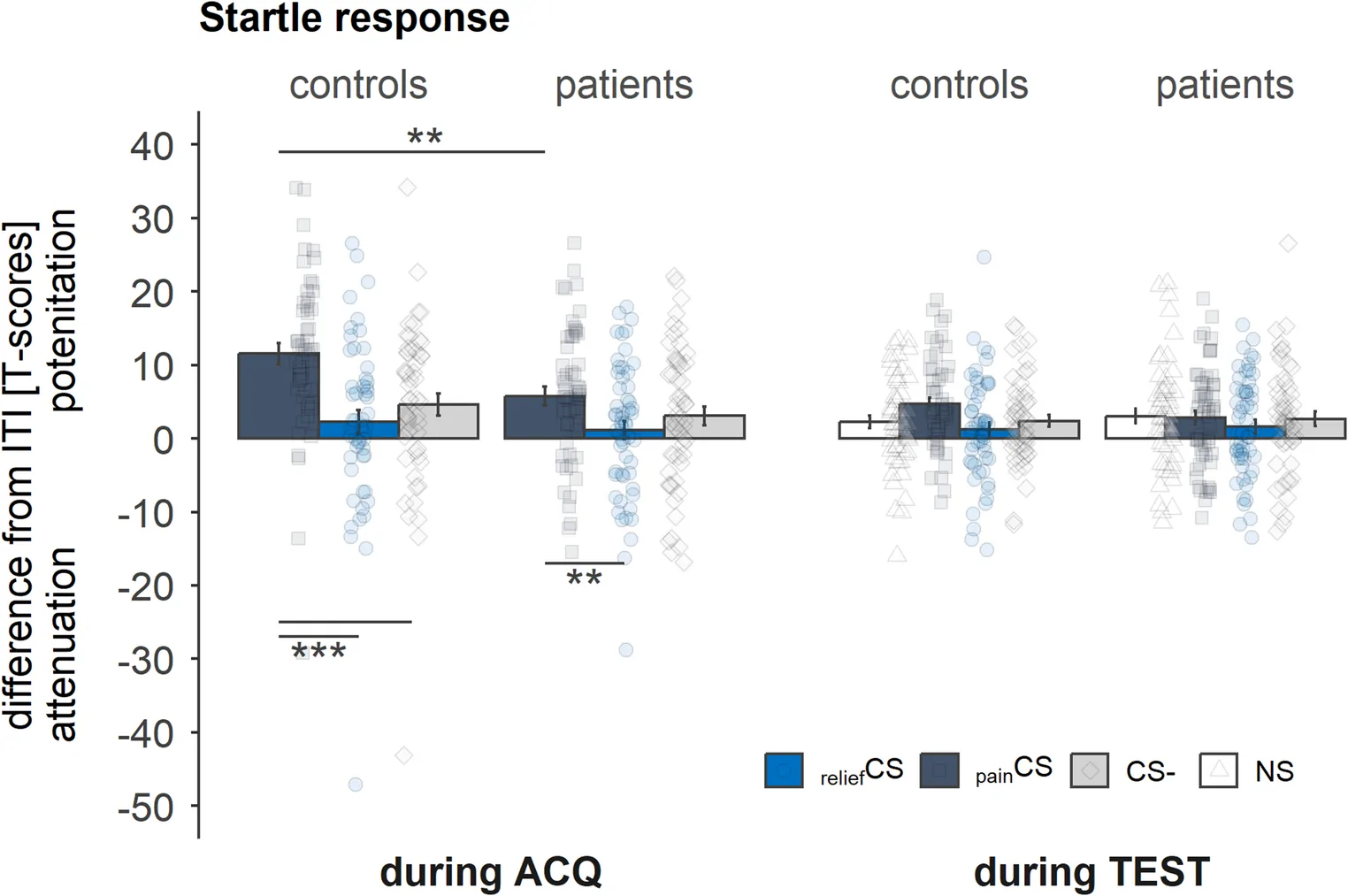
*Group differences in the startle responses*. Bars depict means (with standard errors) during the acquisition blocks (ACQ) and the test blocks (TEST). Adolescent patients showed less discriminative startle responses as the responses to _pain_CS (dark-blue bars and squares) did not differ from CS- (grey bars and diamonds), while relief acquisition was clear in both groups as suggested by the significant response reduction to the _relief_CS (light-blue bars and circles). Adolescent patients however exhibited a blunted startle potentiation to the pain cue as compared to healthy controls. *** *p* < 0.001; ** *p* < 0.01 (after Bonferroni correction)

The significant Stimulus x Group (*F*(2, 106) = 3.76, *p* = 0.026, partial η^2^ = 0.066, Fig. 3) suggests that startle responses of the two groups differed during the acquisition blocks, while no difference were revealed during test (all *p* values > 0.161).

During acquisition, defensive startle responses to _pain_CS were significantly less pronounced for patients than the healthy adolescents (*t*(53) = 2.86, *p* = 0.006, *d* = 0.23). Both groups showed startle potentiation to _pain_CS as compared to _relief_CS (patients: *t*(27) = 3.47, *p* = 0.005, *d* = 0.66; healthy: *t*(26) = 6.42, *p* < 0.001, *d* = 1.24). Like the ratings, learning effects were less pronounced for the adolescent patients than the healthy adolescents. Discriminative startle responses between _pain_CS and CS- were in fact not significant for patients (*t*(27) = 2.34, *p* = 0.081, *d* = 0.44), but for healthy adolescents (*t*(26) = 4.60, *p* < 0.001, *d* = 0.89).

**Fig. 4:**
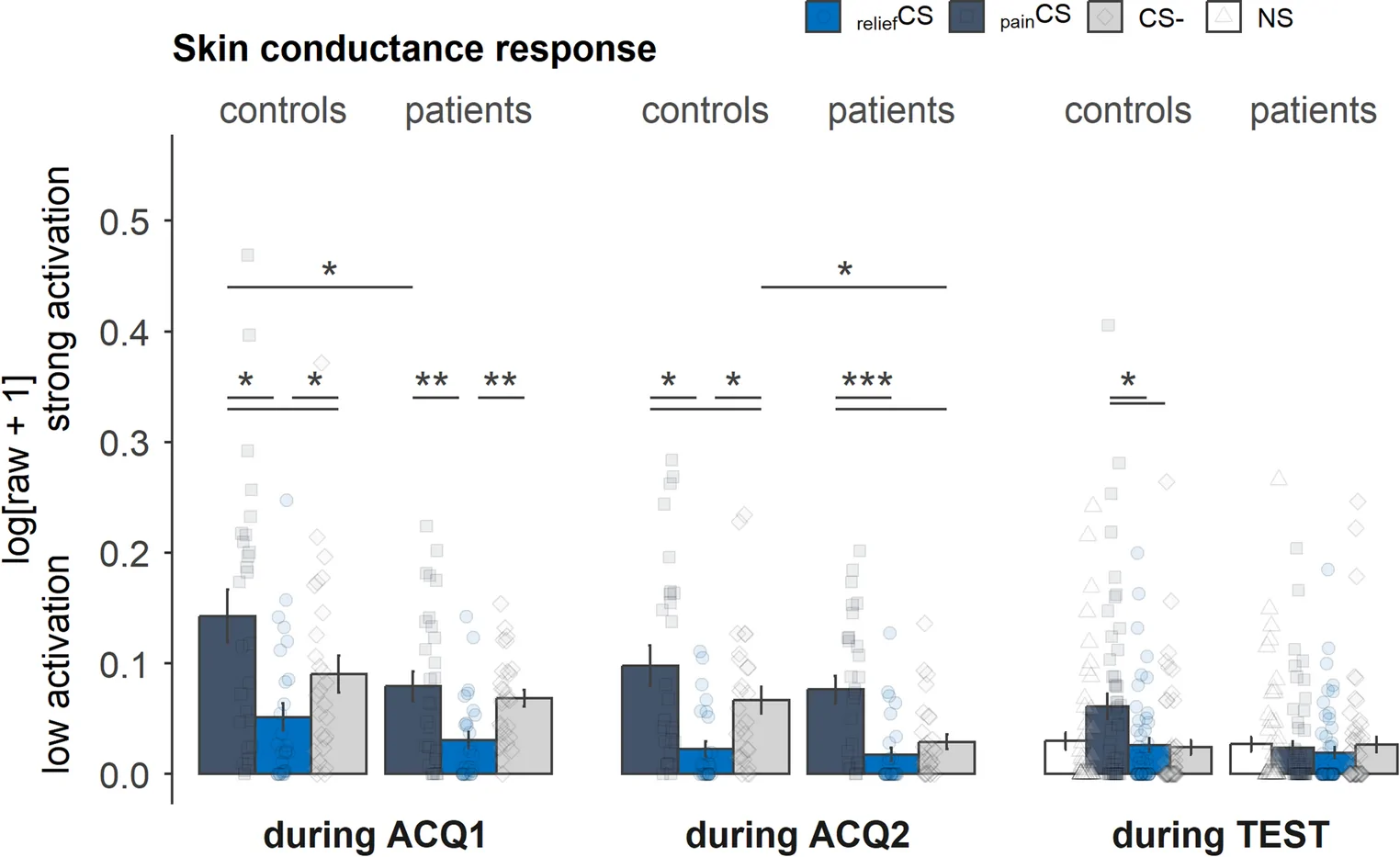
*Group differences in the skin conductance responses*. Bars depict means (with standard errors) during the acquisition blocks (ACQ1 and ACQ2) and the test blocks (TEST). Adolescent patients showed less discriminative SCRs as the responses to _pain_CS (dark-blue bars and squares) did not differ from CS- (grey bars and diamonds) during the first acquisition block, while these discriminative responses became evident during the second acquisition block. Relief acquisition was clear in both groups as the significant reduced responses to the _relief_CS (light-blue bars and circles) suggest. Adolescent patients however presented blunted startle potentiation to the pain cue as compared to healthy controls. *** *p* < 0.001; ** *p* < 0.01; * *p* < 0.05 (after Bonferroni correction)

Similar reduced defensive responses were observed for the SCR as the Stimulus x Phase x Group interaction (*F*(2, 106) = 4.44, *p* = 0.014, partial η^2^ = 0.077, Fig. 4; the post-hoc analyses are reported in Table 3) turned out significant for the acquisition phase and for the test phase the Stimulus x Group interaction (*F*(3, 159) = 4.15, GG-ɛ = 0.794, *p* = 0.013, partial η^2^ = 0.073; the post-hoc analyses are reported in Table 3, column “Test”).

**Table 3:** Post-hoc t-tests for the skin conductance responses

	Adolescent Patients			Healthy Adolescents		
	Acq. Block 1	Acq. Block 2	Test	Acq. Block 1	Acq. Block 2	Test
_pain_CS vs. _relief_CS	** *t*(27) = 3.49, *p* = 0.005, *d* = 0.66	*** *t*(27) = 5.36, *p* < 0.001, *d* = 1.01	*t*(27) = 0.64, *p* > 0.999, *d* = 0.12	*** *t*(26) = 4.46, *p* < 0.001, *d* = 0.86	*** *t*(26) = 4.96, *p* < 0.001, *d* = 0.95	** *t*(26) = 3.62, *p* = 0.008, *d* = 0.70
_pain_CS vs. CS-	*t*(27) = 0.89, *p* > 0.999, *d* = 0.17	*** *t*(27) = 4.26, *p* < 0.001, *d* = 0.81	*t*(27) = 0.31, *p* > 0.999, *d* = 0.06	* *t*(26) = 3.14, *p* = 0.013, *d* = 0.60	* *t*(26) = 2.62, *p* = 0.044, *d* = 0.50	* *t*(26) = 3.12, *p* = 0.026, *d* = 0.60
_relief_CS vs. CS-	** *t*(27) = 3.75, *p* = 0.003, *d* = 0.71	*t*(27) = 1.46, *p* = 0.471, *d* = 0.28	*t*(27) = 0.83, *p* > 0.999, *d* = 0.16	* *t*(26) = 2.78, *p* = 0.030, *d* = 0.54	*** *t*(26) = 4.17, *p* < 0.001, *d* = 0.80	*t*(26) = 0.30, *p* > 0.999, *d* = 0.06
_pain_CS vs. NS	---	---	*t*(27) = 0.37, *p* > 0.999, *d* = 0.07	---	---	*t*(26) = 2.62, *p* = 0.087, *d* = 0.50
_relief_CS vs. NS	---	---	*t*(27) = 0.95, *p* > 0.999, *d* = 0.18	---	---	*t*(26) = 0.47, *p* > 0.999, *d* = 0.09
CS- vs. NS	---	---	*t*(27) = 0.10, *p* > 0.999, *d* = 0.02	---	---	*t*(26) = 0.71, *p* > 0.999, *d* = 0.14

Like the ratings, adolescent patients demonstrated blunted defensive responses as their physiological activation to _pain_CS was significantly lower than healthy adolescents (*t*(53) = 2.35, *p* = 0.023, *d* = 0.63, during acquisition Block1). Adolescent patients also demonstrated differences in their physiological activation to the CS- meaning that patients had significant lower SCRs to CS- than the healthy adolescents (*t*(53) = 2.75, *p* = 0.008, *d* = 0.74) during acquisition Block2. 

During acquisition, SCRs was larger to _pain_CS as compared to _relief_CS for adolescent patients and healthy controls. Adolescent patients needed more learning trials than controls to show discriminative SCRs between _pain_CS and CS-. Discriminative reposes between _relief_CS and CS- were evident during the first acquisition block in both groups. In contrast to healthy adolescents, adolescent patients did not discriminate between _relief_CS and CS- during acquisition Block2. 

Bonferroni corrected *t*-tests during test phase demonstrated larger SCRs to the _pain_CS vs. both the _relief_CS and the CS- for the healthy adolescents, while patients did not show discriminative responses. Again, such reduced learning effect seems to be linked to the blunted responses to the _pain_CS of the patients vs. the controls (*t*(53) = 2.76, *p* = 0.008, *d* = 0.75).

In summary, adolescent patients suffering from NSSI behaviors showed blunted physiological defensive responses to the pain-associated stimulus as compared to the other stimuli.

## Discussion

This study examined whether adolescent patients with NSSI differ in relief-based learning from healthy adolescents. Our results do not support these hypotheses as ratings and physiological responses to the relief-associated cue did not differ between adolescents with and without NSSI. However, we found evidence for blunted physiological and subjective discriminative defensive responses in adolescent patients with NSSI.

This unexpected similarity in the relief response may be explained by the similar intensity and aversiveness of the painful stimulation for both groups. Although we choose a short electro-cutaneous stimulation based on earlier investigations [17, 18], those studies did not report whether the intensity or the ratings of the electric stimulus differ between their two groups making further interpretation of our results difficult. Meta-analyses indicated that patients with NSSI often exhibit higher pain threshold and longer pain tolerance compared to healthy individuals [35], findings that contrast with our results. One explanation may lie in the pain modality. While most studies used thermal or mechanical stimuli lasting several seconds, our electric stimulation lasted only milliseconds engaging in different nociceptive pathways [62]. Our stimulation has most likely captured Abeta-, Adelta-, and certain C-fibers but not fibers associated with chronic pain like CMechanical insensitive fibers (also called silent nociceptors). Thus, our stimulus may not have adequately captured the affective quality of prolonged NSSI-related relief [51, 53]. The unpleasantness of a painful stimulus is primarily encoded in the anterior cingulate cortex, insula, and amygdala and these brain circuits tend to be engaged by prolonged or tonic pain [6]. Whether adaptation of certain fibers like “silent nociceptors” contribute to this phenomenon is currently discussed in the field [29, 56].

The comparable intensity and painfulness of the electro-cutaneous stimulation may also explain the group differences in learned responses. In fact, adolescent patients showed blunted physiological responses and subjective ratings of the pain-predicting stimulus compared to healthy adolescents. Such blunted defensive responses were evident in a reduced or even absent discrimination between the _pain_CS and the CS- as well as in lower responses to the _pain_CS. These reduced defensive responses are consistent with imaging studies showing blunted amygdala activation in response to self-injuries images [28] and aberrant amygdala-frontal connectivity to pain stimuli [65]. It is possible that through repeated exposure to self-inflicted pain, defensive mobilization becomes downregulated, even when conscious awareness of pain remains intact. The divergence between comparable pain sensation as healthy peers but reduced learned defensive responses suggests therefore a decoupling of pain perception and affective-motivational processes in adolescent patients with NSSI. It is possible that repeated self-inflicted pain may lead to a downregulation of defensive responses.

Repeated pain applications can lead to habituation and reduced pain perception [8, 55]. This has been proposed as a risk mechanism for NSSI [21, 30]. However, our findings do not support this hypothesis, as both groups reported similar levels of pain. Learned defensive responses were also not associated with the disease history (i.e., number of NSSI episodes or disorder duration). Consistent with this, previous work [18] reported heightened relief sensitivity that was independent of NSSI frequency. Taken together, these findings suggest distinct alterations in pain- and relief-related processing. Specifically, learning to engage defensive mobilization appears reduced (as indicated by the blunted learned defensive response in our study), alongside with an enhanced relief sensitivity (which was however in your sample independent from NSSI frequency), which may amplify the reinforcing values of self-injuries. Future studies should further elucidate these mechanisms by more clearly disentangling emotional and physical pain and relief experiences [57].

The mean age of our sample was approximately 15 years, placing them in the midst of adolescents. Puberty involves considerable inter-individual variability in onset, duration, and hormonal dynamics [22]. Cortical and sub-cortical regions are still developing [13, 22] and the volume as well as the activity of prefrontal cortex, hippocampus and amygdala are greatly influenced by the hormonal level or by the tempo of puberty [27]. It is therefore plausible that the observed alteration in the defensive responses might be related to developmental processes.

To our knowledge, this study investigated for the first time both pain- and relief-learning in adolescent patients with NSSI. However, some limitations need to be considered. As already discussed, the brief electro-cutaneous stimulation may not have been the most suitable method for modeling the NSSI-related relief. Future studies should apply longer pain stimulations to better model the pathological mechanisms given that cues predicting relief from a thermal pain elicit a strong striatal activation [58]. Second, we applied a classical conditioning paradigm in which two cues (one aversive and one initially neutral) are associated. However, NSSI behaviors are self-administrated self-injuries which may instead involve response-outcome learning [i.e., instrumental learning, 36]. Future studies should therefore examine relief-based instrumental learning. Third, we observed differences in pain intensity and ratings between the participants collected before and after the COVID-19 pandemic. Healthy adolescents showed stronger defensive physiological responses with increasing the stimulation’s intensity, while adolescent patients more strongly attenuated their startle response. These positive-like physiological responses were paralleled by the rating meaning that the pain-predicting stimulus was rated more positively as the subjective pain increased. These differences suggest that adolescent patients might experience intense pain as pleasant supporting our discussion of a decoupling between pain perception and affective-motivational processes. Fourth, all participants were girls. Epidemiological studies outlined that the prevalence of NSSI behaviors is higher in females than in males [49, 53]. However, females and males differ in their self-injury methods as well as in the injured body parts [61]. For a better generalizability of these results, future studies should consider male adolescent patients. Fifth, we did not systematically record whether patients were treated as inpatients or outpatients at the time of participation, which may be relevant for interpreting the findings given potential differences in clinical severity across treatment settings. Sixth, we did not asses comorbid disorder or controlled for depression. Depressive symptoms may have a dampening effect on the physiological responses [47] or alter learning [31]. Alterations due to comorbid disorders could be better control in future studies by collecting adolescent patients without NSSI as control group.

## Conclusions

Our study demonstrated that adolescent patients with NSSI have blunted defensive responses to a stimulus predicting pain, while responses to relief-associated cue were comparable. It is possible that over time repeated self-inflicted pain downregulates discriminative defensive resources. The short painful electro-cutaneous stimulation may have not perceived by the patients as threatening as by healthy adolescents and it was too short to model relief-related mechanisms of NSSI behaviors. While the present findings do not allow direct clinical conclusions, they may inform future research on how altered pain anticipation and relief-related processes in girls contribute to the maintenance of NSSI, and how adaptive relief experiences could be systematically investigated in future experimental and intervention studies — for instance within mindfulness-based or exposure-based strategies.

## Supplementary Information

Below is the link to the electronic supplementary material.

Supplementary Material 1.

## Data Availability

No datasets were generated or analysed during the current study.

## Footnotes

1 Of the six patients with BPD, three met 8 out of 9 diagnostic criteria, two of them met 7 criteria and one met 5 criteria.
