# Supplementary material for: Relief and blunted defensive conditioned responses in girls with non-suicidal self-injury behaviors
Source: Eur Child Adolesc Psychiatry. 2026 Apr 10;35(8):2487–99. doi: 10.1007/s00787-026-03021-7 (PMC13652936; doi:10.1007/s00787-026-03021-7)
Supplement: Supplementary file 1 [file 787_2026_3021_MOESM1_ESM.docx]

Relief and blunted defensive conditioned responses in girls with non-suicidal self-injury behaviors

Marta Andreatta, Nora Banaschewski, Heike Rittner, Marcel Romanos, Arne Bürger, Lorenz Deserno

**Supplementary analyses**

The learning effects as reflected in the within-subject factors stimulus and phase as well as their interaction across both groups are first reported. Moreover, we exploratively examined differences in the unconditioned responses meaning the skin conductance responses to the electric stimulation during acquisition. The preprocessing of the responses has been described in the main text (*2.5 Data reduction*). After the log-transformation, responses were averaged across two trials. Analysis of the variance was calculated having the within-subject factor trial (t1, t2, t3, t4, t5, t6) and the between-subjects factor group (patients, controls).

Because of the pandemic, we collected an initial sample of 31 participants (patients *N* = 20; controls *N* = 11) in 2019 and terminated the data collection in 2023 adding 39 participants (patients *N* = 19; controls *N* = 20). We therefore tested whether the samples of the two data collections differed in any of the demographic or clinical data. Data and comparisons are reported in *Supplementary Table 1*.

**Supplementary results**

*Learning effects across both groups*

*Subjective Ratings*

Briefly, aversive learning was successful as indicated by higher arousal ratings, lower valence ratings, higher expectancy of the US for _pain_CS as compared to the other stimuli. This learned responses remained so even at the end of the test phase suggesting no extinction. On the verbal level the _relief_CS was rated comparably to the CS- and the control stimulus.

The main effects for stimulus (valence: *F*(3, 159) = 9.70, GG-ɛ = 0.890, *p* < 0.001, partial η^2^ = 0.155; arousal: *F*(3, 159) = 19.35, GG-ɛ = 0.879, *p* < 0.001, partial η^2^ = 0.267; US-expectancy: *F*(3, 159) = 108.61, GG-ɛ = 0.694, *p* < 0.001, partial η^2^ = 0.672) and its interaction with phase (***Supp. Fig. 1***; valence: *F*(9, 477) = 8.13, GG-ɛ = 0.592, *p* < 0.001, partial η^2^ = 0.133; arousal: *F*(9, 477) = 22.95, GG-ɛ = 0.664, *p* < 0.001, partial η^2^ = 0.302; US-expectancy: *F*(9, 477) = 10.74, GG-ɛ = 0.559, *p* < 0.001, partial η^2^ = 0.168) turn out significant for all ratings. The main effect for phase was significant for the arousal (*F*(3, 159) = 3.71, GG-ɛ = 0.713, *p* = 0.025, partial η^2^ = 0.065) but not for valence ratings (*F*(3, 159) = 1.02, *p* = 0.384, partial η^2^ = 0.019) and US-expectancy (*F*(3, 159) = 2.36, GG-ɛ = 0.797, *p* = 0.088, partial η^2^ = 0.043).

Bonferroni corrected post-hoc *t*-tests for the 2-way interactions were calculated separately for each block. No difference across the visual stimuli was revealed at the beginning of the experiment, that is after the habituation phase (valence: all *p* values > 0.247; arousal: all *p* values > 0.365).

Affective ratings demonstrated discriminative responses as the _pain_CS was rated more arousing (_relief_CS: *t*(54) = 8.53, *p* < 0.001, *d* = 1.15; CS-: *t*(54) = 8.41, *p* < 0.001, *d* = 1.13; NS: *t*(54) = 10.05, *p* < 0.001, *d* = 1.36) and negatively valenced (_relief_CS: *t*(54) = 4.20, *p* < 0.001, *d* = -0.57; CS-: *t*(54) = 4.61, *p* < 0.001, *d* = -0.62; NS: *t*(54) = 5.15, *p* < 0.001, *d* = -0.69) than the other cues at the end of the acquisition phase (i.e., after the second acquisition block). Participants also reported lower subjective arousal for the NS than the _relief_CS (*t*(54) = 3.05, *p* = 0.021, *d* = 0.41) and the CS- (*t*(54) = 2.87, *p* = 0.035, *d* = 0.39). These discriminative ratings were reflected in the US-expectancies as well. In fact, the painful US was expected significantly more by the _pain_CS as compared to the other cues after both the first (_relief_CS: *t*(54) = 10.18, *p* < 0.001, *d* = 1.37; CS-: *t*(54) = 9.09, *p* < 0.001, *d* = 1.23; NS: *t*(54) = 11.75, *p* < 0.001, *d* = 1.58) and the second (_relief_CS: *t*(54) = 14.49, *p* < 0.001, *d* = 1.95; CS-: *t*(54) = 15.27, *p* < 0.001, *d* = 2.06; NS: *t*(54) = 18.59, *p* < 0.001, *d* = 2.51) acquisition block, while no differences were found between _relief_CS, CS-, and NS (all *p* values > 0.205).

At the end of the first test block, all young participants still expected the aversive US more by _pain_CS than the other stimuli (_relief_CS: *t*(54) = 7.35, *p* < 0.001, *d* = 0.99; CS-: *t*(54) = 7.10, *p* < 0.001, *d* = 0.96; NS: *t*(54) = 7.50, *p* < 0.001, *d* = 1.01), and they reported this stimulus as more arousing (_relief_CS: *t*(54) = 6.30, *p* < 0.001, *d* = 0.85; CS-: *t*(54) = 4.27, *p* < 0.001, *d* = 0.58; NS: *t*(54) = 4.65, *p* < 0.001, *d* = 0.63) and negatively valenced (_relief_CS: *t*(54) = 3.43, *p* = 0.007, *d* = -0.46; CS-: *t*(54) = 2.65, *p* = 0.063, *d* = -0.36; NS: *t*(54) = 3.46, *p* = 0.003, *d* = -0.47). No difference was detected across _relief_CS, CS- and NS (all *p* values > 0.433).

At the end of the second test block, the _pain_CS remained more arousing (*t*(54) = 3.96, *p* = 0.001, *d* = 0.53) and negative (*t*(54) = 3.74, *p* = 0.003, *d* = -0.50) than _relief_CS and participants expected the US more than to the other cues (_relief_CS: *t*(54) = 8.38, *p* < 0.001, *d* = 1.13; CS-: *t*(54) = 7.55, *p* < 0.001, *d* = 1.02; NS: *t*(54) = 7.69, *p* < 0.001, *d* = 1.04). Affective discriminative ratings were however extinguished demonstrated by comparable ratings between the _pain_CS and CS- (arousal: *t*(55) = 1.95, *p* = 0.336, *d* = 0.26; valence: *t*(54) = 1.37, *p* = 1, *d* = -0.18) or NS (arousal: *t*(55) = 2.35, *p* = 0.133, *d* = 0.32; valence: *t*(54) = 1.84, *p* = 0.430, *d* = -0.25).

**Supplementary Figure 1**. *Learning effects for* (**a**.) *US-expectancy*, (**b**.) *arousal and* (**c**.) *valence ratings*. Participants expected the US more by _pain_CS (dark blue lines with s.e.) than the other stimuli after each experimental block (ACQ1: first acquisition block; ACQ2: second acquisition block; TEST1: first test block; TEST2: second test block). Similarly, _pain_CS was rated as more arousing and negatively valenced than _relief_CS (light blue lines with s.e.), CS- (grey lines with s.e.) and the NS (dotted lines with s.e.). _relief_CS remained less arousing and more positive than _pain_CS throughout the test phase. Curves depict the ratings density separately for each condition and phase. For depicting purposes, we subtracted 5 from the valence ratings. *** *p* < 0.001, ** *p* < 0.01

*Physiological responses*

Physiological responses confirmed the learning effects observed on the verbal level. In other words, aversive learning was successful as indicated by startle potentiation and larger SCRs to _pain_CS as compared to the other stimuli. Like the ratings, we found not extinction of the defensive responses (i.e., throughout the test startle remained potentiated and SCRs larger to _pain_CS). Differently from the ratings, physiological responses confirmed relief-like conditioned response as the startle responses and the SCRs were significantly attenuated to _relief_CS as compared to CS-.

Bonferroni corrected *t*-tests for the main effect stimulus (***Supp. Fig. 2***; startle: *F*(2, 106) = 29.01, *p* < 0.001, partial η^2^ = 0.354; SCR: *F*(2, 106) = 36.68, GG-ɛ = 0.783, *p* < 0.001, partial η^2^ = 0.409) revealed that startle responses were potentiated and SCRs were larger to _pain_CS as compared to both _relief_CS (startle: *t*(54) = 6.76, *p* < 0.001, *d* = 0.91; SCR: *t*(54) = 6.91, *p* < 0.001, *d* = 0.93) and CS- (startle: *t*(54) = 4.88, *p* < 0.001, *d* = 0.66; SCR: *t*(54) = 4.61, *p* < 0.001, *d* = 0.62). Relief-related responses were confirmed by the attenuation of the startle response (*t*(54) = 2.48, *p* = 0.049, *d* = -0.33) as well as the lower SCR (*t*(54) = 5.32, *p* < 0.001, *d* = 0.72) to the _relief_CS than to CS-. Despite physiological responses habituated throughout the two acquisition blocks (main effect phase: [startle] *F*(1, 53) = 4.91, *p* = 0.031, partial η^2^ = 0.085; [SCR] *F*(1, 53) = 24.31, *p* < 0.001, partial η^2^ = 0.314), this habituation process was not different between the stimulus (interaction Stimulus x Phase [startle] *F*(2, 106) = 2.10, *p* = 0.127, partial η^2^ = 0.038; [SCR] *F*(2, 106) = 0.62, *p* = 0.538, partial η^2^ = 0.012).

During the test phase, the main effect stimulus (***Supp. Fig. 2***) remained significant for both startle responses (*F*(3, 159) = 4.49, *p* = 0.005, partial η^2^ = 0.078) and SCRs (*F*(3, 159) = 3.99, GG-ɛ = 0.783, *p* = 0.015, partial η^2^ = 0.070), but not the main effect phase (startle: *F*(1, 53) = 2.54, *p* = 0.117, partial η^2^ = 0.046; SCR: *F*(1, 53) = 2.57, *p* = 0.115, partial η^2^ = 0.046) or their interaction (startle: *F*(3, 159) = 0.30, *p* = 0.825, partial η^2^ = 0.006; SCR: *F*(3, 159) = 0.55, *p* = 0.646, partial η^2^ = 0.010). Startle responses (*t*(54) = 3.31, *p* = 0.010, *d* = 0.45) and SCRs (*t*(54) = 2.13, *p* = 0.017, *d* = 0.42) remained significantly potentiated to _pain_CS as compared to _relief_CS throughout test blocks.

**Supplementary Figure 2**. *Main effect stimulus* *for* (**a**.) *startle responses and* (**b**.) *skin conductance responses* separately for each phase. During acquisition phase, startle responses were potentiated and SCRs were larger to the _pain_CS (dark blue bars and squares [with s.e.]) than the _relief_CS (light blue bars and circles [with s.e.]) and the CS- (grey bras and diamonds [with s.e.]). Relief responses were evident as the physiological responses were attenuated to the _relief_CS as compared to CS-. During test, _pain_CS still elicited stronger startle responses and larger SCRs than _relief_CS. White bars and triangles (with s.e.) depict the responses to the NS. *** *p* < 0.001, ** *p* < 0.01, * *p* < 0.050

*Unconditioned responses*

ANOVA returned a significant main effect trial (*F*(5, 265) = 5.75, GG-ɛ = 0.819, *p* < 0.001, partial η^2^ = 0.098), while the main effect group (*F*(1, 53) = 3.18, *p* = 0.080, partial η^2^ = 0.057) and its interaction with trial (*F*(5, 265) = 1.92, GG-ɛ = 0.819, *p* = 0.091, partial η^2^ = 0.035) just failed to reach the significance level. The main effect trial revealed habituation of the skin conductance response to the aversive stimulation through the learning trials (see ***Supp. Fig. 3***). Although adolescent patients (*M*: 0.26; *SD*: 0.14) had a slightly higher physiological arousal than controls (*M*: 0.20; *SD*: 0.12), habituation of the SCRs was comparable between the two groups.

**Supplementary Figure 3**. S*kin conductance responses to the electric stimulation*. Lines (with *s.e.*) depict SCRs to US separately for controls (dashed viola lines and circles) and patients (solid yellow lines and squares). During acquisition phase, SCRs to the US significantly habituated but no group difference was revealed.

**Supplementary discussion**

The pain-eliciting stimulus used in this study was a short electro-cutaneous stimulation, which has commonly used in learning protocols in healthy adults [13] and patients [9]. Adolescents of this study exhibited startle potentiation, heightened SCRs, more aversive ratings and higher US-expectancies to the pain-predicting CS compared to the CS-. This is in line with the results in healthy adults [2, 20, 22, 24] and it replicates earlier results in children [for a review see, 4, 8, 18, 23], in adolescents [19] and in mixed samples of children and adolescents [17, 25]. Together, these findings suggest that aversive learning is readily acquired even at young age, possibly due to its evolutionary relevance for survival.

The cue (i.e., _relief_CS) presented after the termination of the short painful US induced a pleasant feeling of relief as suggested by the attenuation of the physiological responses, which parallels the responses in adults [2, 6, 7]. The observed discriminative responses between _relief_CS and CS- aligned animal findings, which point to a distinction between responses elicited by pain termination (i.e., relief) and those elicited by pain absence [i.e., safety, 14, 15]. To our knowledge, no study has yet investigated relief-learning in young populations or compared such responses between younger individuals and adults. Nevertheless, we think that the observed results reflect the same evolutionary relevance attributed to the pain-related learning. Relief can in fact be conceptualized as a part of a continuum, with pain at one end and its absence at the other [12]. As opponent-reaction to pain, relief is an automatic response triggered by the cessation of an aversive event [11, 16, 21] and relief learning might therefore represent a form of primary learning rooted in adaptive survival mechanisms.

Verbal reports diverged from physiological responses demonstrating comparable valence, arousal or US-expectations for both the relief-associated stimulus (_relief_CS) and the safety signal (CS-), which confirms previous studies [1, 2]. This dissociation suggests that verbal and physiological responses may have distinct underlying mechanisms [3, 10]. When an aversive event is signaled by a cue (i.e., _pain_CS), this pain-predicting cue holds the greatest informational value regarding the occurrence of the pain, and can therefore cognitively be prioritized and evaluated as aversive. On the other hand, the cessation and the absence of an aversive event appear cognitively equivalent as both cues signal no immediate pain.

**Comparisons between the two data collections**

| ***Supplementary Table 1.*** *Descriptive statistics*. Comparisons between the two data collections. | | | | |
| --- | --- | --- | --- | --- |
|  | **Patients** | | **Controls** | |
|  | *2019* | *2023* | *2019* | *2023* |
| *N (BPS)* | 13 (2) | 15 (4) | 10 | 17 |
| age (*sd*) | 16.37 (0.98) | 15.43 (1.41) | 15.87 (1.41) | 15.37 (1.66) |
| mA (*sd*) | 1.96 (0.69) | 3.78 (1.91) | 2.11 (0.38) | 2.63 (0.42) |
| painful (*sd*) | 6.97 (1.72) | 5.29 (1.35) | 6.55 (1.25) | 6.88 (1.81) |
| STAI X2 (*sd*) | 62.00 (6.28) | 61.60 (7.74) | 33.50 (5.84) | 38.29 (9.94) |
| SCL90 (*sd*) | --- | --- | 0.30 (0.36) | 0.65 (0.54) |

The 2 (group: patients, controls) x 2 (year: 2019, 2023) ANOVA returned no significant difference for age. Descriptively, individuals collected in 2019 were slightly older (*M*: 16.15, *sd*: 1.18) than those collected in 2023 (*M*: 15.40, *sd*: 1.53; main effect year: *F*(1, 51) = 3.42, *p* = 0.070, partial η^2^ = 0.063), but there was no main effect of group (*F*(1, 51) = 0.52, *p* = 0.475 partial η^2^ = 0.010) or interaction effect (*F*(1, 51) = 0.32, *p* = 0.575, partial η^2^ = 0.006).

The 2 (group: patients, controls) x 2 (year: 2019, 2023) ANOVA for the STAI trait (X2) returned significant higher trait anxiety for the patients (*M*: 61.79, *sd*: 6.98) as compared to the controls (*M*: 36.52, *sd*: 8.84; main effect group: *F*(1, 51) = 141.46, *p* < 0.001, partial η^2^ = 0.735), while the main effect year (*F*(1, 51) = 1.02, *p* = 0.318, partial η^2^ = 0.020) or their interaction (*F*(1, 51) = 1.42, *p* = 0.239, partial η^2^ = 0.027) were not significant. Comparisons for the healthy controls of the two data collection returned no significant differences (*t*(23) = 1.65, *p* = 0.112, *d* = 0.43) meaning that controls were comparable healthy and the low SCL90 score confirm the absence of symptoms. We calculated *t*-tests or Chi-quadrat for the items of the SITBI-G questionnaire [5] and for an overview of the methods used, see *Supplementary Table 3*.

| ***Supplementary Table 2.*** *Methods used for NSSI behaviors.* | | | |
| --- | --- | --- | --- |
|  | *2019* | *2023* | *comparisons* |
| *Method* | *n* (%) | *n* (%) |  |
| cut or carved skin | 13 (100) | 14 (93.3) | χ^2^(1) < 0.01, *p* = 1 |
| hit yourself on purpose | 11 (84.6) | 9 (60) | χ^2^(1) = 3.69, *p* = 0.055 |
| pulled your hair out | 4 (30.8) | 6 (40) | χ^2^(1) = 2.47, *p* = 0.116 |
| gave yourself a tattoo | 1 (7.7) | 1 (6.7) | χ^2^(1) = 1.37, *p* = 0.242 |
| picked at a wound | 10 (76.9) | 10 (66.7) | χ^2^(1) = 2.34, *p* = 0.126 |
| burned your skin (i.e., with a cigarette, match or other hot object) | 6 (46.2) | 8 (53.3) | χ^2^(1) = 2.36, *p* = 0.124 |
| inserted objects under your nails or skin | 1 (7.7) | 2 (13.3) | χ^2^(1) = 0.68, *p* = 0.408 |
| bit yourself (e.g., your mouth or lip) | 5 (38.5) | 7 (46.7) | χ^2^(1) = 0.250, *p* = 0.114 |
| picked areas of your body to the point of drawing blood | 2 (15.4) | 8 (53.3) | χ^2^(1) = 0.24, *p* = 0.621 |
| scraped your skin | 8 (61.5) | 11 (73.3) | χ^2^(1) = 1.06, *p* = 0.303 |
| “erased” your skin to the point of drawing blood | 4 (30.8) | 8 (53.3) | χ^2^(1) = 1.29, *p* = 0.256 |

The 2 (group: patients, controls) x 2 (year: 2019, 2023) ANOVA for the US returned higher intensity of the electric stimulation for those participants collected in 2019 (*M*: 2.03, *sd*: 0.57) as compared to those collected in 2023 (*M*: 3.17, *sd*: 1.44; main effect year: *F*(1, 51) = 15.25, *p* < 0.001, partial η^2^ = 0.230). While the main effect group failed to reach the significance level (*F*(1, 51) = 2.83, *p* = 0.099, partial η^2^ = 0.053), the interaction between group and year was significant (*F*(1, 51) = 4.62, *p* = 0.036, partial η^2^ = 0.083). The Group x Year interaction turned out significant also for the pain ratings (*F*(1, 51) = 7.06, *p* = 0.011, partial η^2^ = 0.122), but not the main effects for group (*F*(1, 51) = 0.37, *p* = 0.547, partial η^2^ = 0.007) and year (*F*(1, 51) = 1.92, *p* = 0.172, partial η^2^ = 0.036). Bonferroni corrected post-hoc *t*-tests revealed no group differences in both data collections, the 2019 (US-intensity: *t*(21) = 0.58, *p* = 0.571, *d* = 0.24; US-rating: *t*(21) = 1.55, *p* = 0.545, *d* = 0.65) and the 2023 (US-intensity: *t*(30) = 2.43, *p* = 0.086, *d* = 0.86; US-ratings: *t*(30) = 2.34, *p* = 0.106, *d* = 0.83). The US intensities for both the patients (*t*(26) = 3.25, *p* = 0.013, *d* = 1.23) and the controls (*t*(25) = 3.27, *p* = 0.012, *d* = 1.30) collected in 2019 were higher than those collected in 2023. Patients (*t*(26) = 3.90, *p* = 0.019, *d* = 1.17), but not controls (*t*(25) = 0.84, *p* = 0.412, *d* = 0.33) collected in 2023 reported significantly less pain than those collected in 2019.

Due to these significant differences, we re-run all the analyses adding the US intensity and the US ratings separately as covariate. Arousal ratings (all *p* values > 0.063) and the US-expectancy (all *p* values > 0.067) were not altered by US intensity. While valence ratings were slightly changed by US intensity as indicated by the significant Group x Stimulus x US intensity interaction (*F*(3, 153) = 2.93, *p* = 0.036, partial η^2^ = 0.054; ***Supp. Fig. 4a***). As post-hoc tests, we calculated Bonferroni-corrected Pearsons’s correlations for the two groups separately and found no significant effects (all *p* values > 0.121).

**Supplementary Figure 4**. *Correlations between US (****a****.) intensity or (****b****.) painful ratings and the valence of _relief_CS*. The more painful the electric stimulation was for the adolescent patients (yellow lines with s.e.) was, the more negative they rated the _relief_CS. US intensity did not change valence ratings. Ratings of the healthy controls (viola lines with s.e.) were not modulated by the US.

Similar to the US intensity, US pain ratings did not modify arousal (all *p* values > 0.121; except for a main effect: *F*(1, 51) = 10.10, *p* = 0.003, partial η^2^ = 0.165) and US-expectancy (all *p* values > 0.073), but valence ratings (main effect: *F*(1, 51) = 6.40, *p* = 0.015, partial η^2^ = 0.112; 4-way interaction: *F*(9, 459) = 2.20, *p* = 0.021, partial η^2^ = 0.041). Again, as post-hoc tests for the interaction Group x Stimulus x Phase x US ratings we calculated Bonferroni-corrected Pearsons’s correlations for the two groups separately, but only for the second acquisition and test blocks. At the end of the acquisition phase (***Supp. Fig. 4b***), adolescents patients rated the _relief_CS more positively valenced, the more painful they reported the US (*r*(26) = 0.53, *p* = 0.014). US pain ratings did not correlate with the other cues (all *p* values > 0.687) or at the end of the second block of the test phase (all *p* values > 0.270). Healthy adolescents, in contrast, did not change their valence rating during the acquisition (all *p* values > 0.935), but reported the _pain_CS at the end of the experiment the more negative, the higher their pain ratings were (*r*(25) = 0.52, *p* = 0.022).

Physiological responses were more pronouncedly impacted by the US intensity than the ratings. In fact, the Group x Stimulus x US intensity (*F*(2, 102) = 4.44, *p* = 0.014, partial η^2^ = 0.080) and the Group x Stimulus x Phase x US intensity (*F*(2, 102) = 3.39, *p* = 0.037, partial η^2^ = 0.062) interactions turned out significant for SCR and startle response, respectively. Bonferroni corrected post-hoc Pearson’s correlation for the 3-way interaction revealed that SCR was not significantly modulated by US intensity (all *p* values > 0.721). Startle responses (***Supp. Fig. 5a***) to CS- during the second acquisition block were significantly attenuated, the more painful the US was reported by the adolescent patients (*r*(26) = -0.47, *p* = 0.034), whereas these responses were significantly potentiated in the healthy controls (*r*(25) = 0.48, *p* = 0.033).

**Supplementary Figure 5**. *Correlations between US (****a****.) intensity or (****b****.) pain ratings and the startle responses during the second acquisition block to CS-*. The more intense the electric stimulation was, the more startle attenuation to CS- was elicited among adolescent patients (yellow lines with s.e.) but the more startle potentiation among the healthy controls (viola lines with s.e.). No effects were found for the pain ratings of the electric stimulation.

During test, the physiological arousal but not the startle response (all *p* values > 0.094) was altered by US intensity (Group x Stimulus x US intensity: *F*(3, 153) = 2.77, *p* = 0.044, partial η^2^ = 0.052). This effect was driven by the control group as their SCRs to both _pain_CS (*r*(25) = -0.54, *p* = 0.010; ***Supp. Fig. 6a***) and _relief_CS (*r*(25) = -0.51, *p* = 0.021) were lower, the more painful the US was reported at the beginning of the experiment.

US pain ratings did not change startle effects during both acquisition (all *p* values > 0.236; ***Supp. Fig. 5b***) and test (all *p* values > 0.111) blocks. SCR (***Supp. Fig. 6***), on the other hand, was changed by these ratings during acquisition (Group x Stimulus x US ratings: *F*(2, 102) = 10.22, *p* < 0.001, partial η^2^ = 0.167) but not during test (all *p* values > 0.387). Bonferroni corrected Pearson’s correlations revealed that for those patients, who reported the electric stimulation painful, the physiological arousal to _pain_CS significantly increased (*r*(26) = 0.62, *p* = 0.001). No other effects were found among the adolescent patients (all *p* values > 0.462) and the controls (all *p* values > 0.270).

**Supplementary Figure 6**. *Correlations between US (****a****.,* ***c****.) intensity or (****b****.,* ***d****.) pain ratings and the skin conductance responses to _pain_CS*. During acquisition blocks, the more painful the electric stimulation was, the physiological arousal to *_pain_CS* was elicited among adolescent patients (yellow lines with s.e.). The more intense the stimulation was, the less physiological arousal to *_pain_CS was elicited* among the healthy controls (viola lines with s.e.) during test blocks.

**References**

1.	Andreatta M, Mühlberger A, Glotzbach-Schoon E, Pauli P (2013) Pain predictability reverses valence ratings of a relief-associated stimulus. Frontiers in Systems Neuroscience 7, doi: 10.3389/fnsys.2013.00053

2.	Andreatta M, Pauli P (2017) Learning mechanisms underlying threat absence and threat relief: Influences of trait anxiety. Neurobiology of Learning and Memory 145:105–113, https://doi.org/10.1016/j.nlm.2017.09.005

3.	Bradley MM, Codispoti M, Lang PJ (2006) A multi-process account of startle modulation during affective perception. Psychophysiology 43:486–497, doi:

4.	Britton JC, Lissek S, Grillon C, Norcross MA, Pine DS (2011) Development of anxiety: the role of threat appraisal and fear learning. Depression and Anxiety 28:5–17, doi: 10.1002/da.20733

5.	Fischer G, Ameis N, Parzer P, Plener PL, Groschwitz R, Vonderlin E, Kölch M, Brunner R, Kaess M (2014) The German version of the self-injurious thoughts and behaviors interview (SITBI-G): a tool to assess non-suicidal self-injury and suicidal behavior disorder. BMC Psychiatry 14:265, doi: 10.1186/s12888-014-0265-0

6.	Gerber B, König C, Fendt M, Andreatta M, Romanos M, Pauli P, Yarali A (2019) Timing-dependent valence reversal: a principle of reinforcement processing and its possible implications. Current Opinion in Behavioral Sciences 26:114–120, https://doi.org/10.1016/j.cobeha.2018.12.001

7.	Gerber B, Yarali A, Diegelmann S, Wotjak CT, Pauli P, Fendt M (2014) Pain-relief learning in flies, rats, and man: basic research and applied perspectives. Learning & Memory 21:232–252, doi: 10.1101/lm.032995.113

8.	Glenn CR, Klein DN, Lissek S, Britton JC, Pine DS, Hajcak G (2012) The development of fear learning and generalization in 8–13 year-olds. Developmental Psychobiology 54:675–684, doi: 10.1002/dev.20616

9.	Kausche FM, Carsten HP, Sobania KM, Riesel A (2025) Fear and safety learning in anxiety- and stress-related disorders: An updated meta-analysis. Neuroscience & Biobehavioral Reviews 169:105983, https://doi.org/10.1016/j.neubiorev.2024.105983

10.	Lang PJ (1995) The emotion probe. Studies of motivation and attention. American Psychologist 50:372–385, doi: https://doi.org/10.1037/0003-066X.50.5.372

11.	Leknes S, Tracey I (2008) A common neurobiology of pain and pleasure. Nature Reviews. Neuroscience 9:314–320, doi:10.1038/nrn2333

12.	Lohr JM, Olatunji BO, Sawchuk CN (2007) A functional analysis of danger and safety signals in anxiety disorders. Clinical Psychology Review 27:114–126, doi: 10.1016/j.cpr.2006.07.005

13.	Lonsdorf TB, Menz MM, Andreatta M, Fullana MA, Golkar A, Haaker J, Heitland I, Hermann A, Kuhn M, Kruse O, Meir Drexler S, Meulders A, Nees F, Pittig A, Richter J, Römer S, Shiban Y, Schmitz A, Straube B, Vervliet B, Wendt J, Baas JMP, Merz CJ (2017) Don’t fear ‘fear conditioning’: Methodological considerations for the design and analysis of studies on human fear acquisition, extinction, and return of fear. Neuroscience & Biobehavioral Reviews 77:247–285, http://dx.doi.org/10.1016/j.neubiorev.2017.02.026

14.	Mayer D, Kahl E, Uzuneser TC, Fendt M (2018) Role of the mesolimbic dopamine system in relief learning. Neuropsychopharmacology 43:1651–1659, doi: 10.1038/s41386-018-0020-1

15.	Mohammadi M, Bergado-Acosta JR, Fendt M (2014) Relief learning is distinguished from safety learning by the requirement of the nucleus accumbens. Behavioural Brain Research 272:40–45, http://dx.doi.org/10.1016/j.bbr.2014.06.053

16.	Navratilova E, Porreca F (2014) Reward and motivation in pain and pain relief. Nat Neurosci 17:1304–1312, doi: 10.1038/nn.3811

17.	Reinhard J, Slyschak A, Schiele MA, Andreatta M, Kneer K, Reif A, Domschke K, Gamer M, Pauli P, Deckert J, Romanos M (2021) Fear conditioning and stimulus generalization in association with age in children and adolescents. European Child & Adolescent Psychiatry, doi: 10.1007/s00787-021-01797-4

18.	Schiele MA, Reinhard J, Reif A, Domschke K, Romanos M, Deckert J, Pauli P (2016) Developmental aspects of fear: Comparing the acquisition and generalization of conditioned fear in children and adults. Developmental Psychobiology 58:471–481, doi: 10.1002/dev.21393

19.	Shechner T, Britton JC, Ronkin EG, Jarcho JM, Mash JA, Michalska KJ, Leibenluft E, Pine DS (2015) Fear conditioning and extinction in axious and nonanxious youth and adults: Examining a novel developmentally appropriate fear-conditioning task. Depression and Anxiety 32:277–288, https://doi.org/10.1002/da.22318

20.	Sjouwerman R, Niehaus J, Kuhn M, Lonsdorf TB (2016) Don't startle me—Interference of startle probe presentations and intermittent ratings with fear acquisition. Psychophysiology 53:1889–1899, doi: 10.1111/psyp.12761

21.	Solomon RL (1980) The opponent-process theory of acquired motivation. The cost of pleasure and the benefit of pain. American Psychologist 35:691–712, https://doi.org/10.1037/0003-066X.35.8.691

22.	Sperl MFJ, Panitz C, Hermann C, Mueller EM (2016) A pragmatic comparison of noise burst and electric shock unconditioned stimuli for fear conditioning research with many trials. Psychophysiology 53:1352–1365, https://doi.org/10.1111/psyp.12677

23.	Stenson AF, Nugent NR, van Rooij SJH, Minton ST, Compton AB, Hinrichs R, Jovanovic T (2020) Puberty drives fear learning during adolescence. Developmental Science 24:e13000, doi: 10.1111/desc.13000

24.	Torrents-Rodas D, Fullana MA, Bonillo A, Caseras X, Andión O, Torrubia R (2013) No effect of trait anxiety on differential fear conditioning or fear generalization. Biological Psychology 92:185–190, http://dx.doi.org/10.1016/j.biopsycho.2012.10.006

25.	Widegren E, Vegelius J, Frick MA, Roy AA, Möller S, Kleberg JL, Hoppe JM, Hjorth O, Fällmar D, Pine DS, Brocki K, Gingnell M, Frick A (2025) Fear extinction retention in children, adolescents, and adults. Developmental Cognitive Neuroscience 71:101509, https://doi.org/10.1016/j.dcn.2025.101509
